# Maternal adverse childhood experiences and preschool children’s behavioral problems: exploring mediation via an adapted measure of adult attachment pattern

**DOI:** 10.3389/fpsyg.2025.1596613

**Published:** 2025-09-02

**Authors:** Stefan Kurbatfinski, Aliyah Dosani, Andrew F. Hayes, Deborah Dewey, Nicole Letourneau

**Affiliations:** 1 Department of Community Health Sciences, Cumming School of Medicine, University of Calgary, Calgary, AB, Canada; 2 Alberta Children's Hospital Research Institute (ACHRI), Owerko Centre, Calgary, AB, Canada; 3 Faculty of Health, Community, and Education, School of Nursing and Midwifery, Mount Royal University, Calgary, AB, Canada; 4 O'Brien Institute for Public Health, Cumming School of Medicine, University of Calgary, Calgary, AB, Canada; 5 Haskayne School of Business, University of Calgary, Calgary, AB, Canada; 6 Hotchkiss Brain Institute, Cumming School of Medicine, University of Calgary, Calgary, AB, Canada; 7 Departments of Community Health Sciences and Pediatrics, Cumming School of Medicine, University of Calgary, Calgary, AB, Canada; 8 Faculty of Nursing and Cumming School of Medicine (Departments of Community Health Sciences, Pediatrics, and Psychiatry), University of Calgary, Calgary, AB, Canada

**Keywords:** adult attachment pattern, mediation and moderation, maternal adverse childhood experiences, children’s behavior, mental health, child sex-assigned-at-birth, Experiences in Close Relationships

## Abstract

**Background:**

Mothers’ insecure adult attachment pattern (i.e., dismissive, preoccupied) has been proposed to positively mediate the associations between mothers’ adverse childhood experiences (ACEs) and their preschool children’s sex-specific behavioral problems. However, findings remain mixed with few focusing on a total score ranging from secure to insecure. Therefore, the aim of this study was to novelly employ the Revised-Experiences in Close Relationships (ECR-R) questionnaire to explore if (1) mothers’ adult attachment pattern measured continuously from secure to insecure mediates the relationship between maternal ACEs and children’s internalizing and externalizing behaviors and (2) child sex-assigned-at-birth moderated the effects.

**Methods:**

Data (*n* = 636) derived from the prospective APrON Study (participants recruited during early pregnancy). Maternal ACEs were measured at child age 1, while children’s behavioral problems and mothers’ adult attachment pattern at child age 5.

**Findings:**

Positive, indirect effects on children’s internalizing (*bootstrap 95% CI [0.10, 0.58]) and externalizing (*[0.11, 0.62]) problems were supported. Post-hoc, a positive, indirect effect through preoccupied adult attachment pattern on their children’s internalizing problems was supported (*[0.04, 0.47]), but the effect did not differ from that of dismissive adult attachment pattern (*[−0.45, 0.15]). No moderation was observed (*p* > 0.05).

**Significance:**

This study is the first to employ the ECR-R to measure mothers’ adult attachment pattern continuously from secure to insecure. Findings suggest that (1) adult attachment pattern measured continuously mediates the association between mothers’ ACEs and their preschool children’s behavioral problems and (2) preoccupied adult attachment pattern behaviors may play a stronger role than those of dismissive.

## Introduction

Adverse childhood experiences (ACEs) are considered to be stressful or traumatic life events experienced by children before 18 years of age (Felitti et al., 1998). These include: physical, sexual, and emotional abuse and/or neglect; parental loss, separation, divorce, or incarceration; and parental substance use and mental illness (Felitti et al., 1998). ACEs can increase individuals’ risk of later health concerns including mental (e.g., depression) and physical (e.g., metabolic, cardiovascular, and immune dysfunction) health problems (Nelson et al., 2020; Madigan et al., 2023). The impacts of ACEs can also occur across generations (Moog et al., 2023; Letourneau et al., 2019). Since mothers are often primary parents and research has predominantly focused on their ACEs and children’s behavioral problems (Arnold et al., 2023), mothers will be referred to throughout the study; however, the intention is not to ignore the important role of fathers who are being increasingly studied (Seteanu and Giosan, 2021; Schickedanz et al., 2018). Research findings consistently show positive associations between mothers’ ACEs and their children’s behavioral problems (Cooke et al., 2021). Importantly, children’s exposure to mothers’ ACEs during the preschool period, a critical time of rapid growth and development, may have especially pronounced impacts (Brown and Jernigan, 2012; Sakai, 2020), making this a key stage for studying predictors of children’s behavioral outcomes. Moreover, male sex-assigned-at-birth (SAAB; sex assignment based on external anatomy and biological factors) children have demonstrated greater vulnerability to maternal ACEs compared to female children (Letourneau et al., 2019; Li et al., 2024). However, the manner through which mothers’ ACEs exert their negative influence on preschool children’s sex-specific behavioral problems is not well understood.

While the intergenerational impacts of mothers’ ACEs on their preschool children’s behavioral problems are posited to operate through suboptimal parenting (Rowell and Neal-Barnett, 2022), findings across studies remain inconsistent (Rowell and Neal-Barnett, 2022; Luo et al., 2023; Madigan et al., 2015), suggesting that other potential mediators should be explored. Mothers’ adult attachment pattern, referring to their perceived relationship with their intimate partner, offers an intriguing possibility for investigating the association between mothers’ ACEs and their preschool children’s behavioral problems (Kurbatfinski et al., 2023), as it is linked to mothers’ early childhood experiences (Silva et al., 2024), the quality of parenting mothers provide (Huang, 2021), and their children’s behavior (Jing and Michiyo, 2023). A recent systematic review examining mothers’ adult attachment pattern as a mediator of the association between mothers’ ACEs and their preschool children’s behavioral problems suggested that this requires more study (Kurbatfinski et al., 2023).

### Adult attachment pattern as a mediator

A secure adult attachment pattern is characterized by safety and consistency in intimate relationships and linked to more positive health outcomes (Bartholomew and Horowitz, 1991). Conversely, insecure adult attachment pattern is linked to worsened health (Mikulincer and Shaver, 2012; Guo and Ash, 2020). Two main insecure adult attachment pattern subtypes are often discussed, including: (1) dismissive adult attachment pattern, which is characterized by adult avoidance of psychologically intimate relationships and (2) preoccupied adult attachment pattern, which is characterized by an adult’s need for copious reassurance and comfort from their intimate partners (Mikulincer and Shaver, 2012). While these two patterns of attachment are typically conceptualized as attachment toward an intimate partner, there is evidence that behaviors related to these attachment patterns can be observed across different types of relationships, including mother–child relationships (Fraley et al., 2011; Shah et al., 2010).

More insecure adult attachment pattern among mothers has been associated with their children’s internalizing and externalizing problems (Jing and Michiyo, 2023), including emotional reactivity, anxiety and depressive symptoms, withdrawal, and aggression (Kohlhoff et al., 2024). Evidence also points to the relevant impacts of fathers’ adult attachment pattern on their children’s behavioral problems (Cowan et al., 1996). Findings from a systematic review conducted by this team found only two studies that examined mothers’ adult attachment patterns as mediators of the association between mothers’ ACEs and their preschool children’s behavioral problems (Kurbatfinski et al., 2023). Of those, one observed mediation only through secure (versus less secure) adult attachment pattern (Roth et al., 2021), while the other reported mediation through preoccupied or both preoccupied and dismissive adult attachment patterns only when maternal depressive symptoms were also included as a sequential mediator (Cooke et al., 2019). Therefore, specifying the role of adult attachment pattern in the association between mothers’ ACEs and their children’s behavioral problems, specifically during the sensitive preschool period, could help in clarifying the relevance of mothers’ ACEs to their children’s behavior and guide interventions for preschool children at-risk of, or exhibiting, behavioral problems.

### Preschool children’s behavioral problems reflect poor mental health

Mental health concerns in children often manifest as behavioral problems, which are subdivided into internalizing and externalizing behaviors (Reynolds and Kamphaus, 2004). Internalizing behaviors (e.g., anxiety, depression, withdrawal) are related to one’s internal psychological state, reflecting mood and emotion (Nikstat and Riemann, 2020). On the other hand, externalizing behaviors (e.g., aggression, hyperactivity) are outwardly expressed and physical in nature, reflecting an inability to cope with stressors (Nikstat and Riemann, 2020). Internalizing behaviors are often more difficult to diagnose due to their more self-directed pattern, whereas externalizing behaviors are more visible, permitting earlier identification (Nikstat and Riemann, 2020). Regardless, without intervention, both internalizing and externalizing problems are associated with suboptimal outcomes later in life, including later mental and physical health concerns and lower socioeconomic attainment (Mulraney et al., 2021). For example, findings from a systematic review of 40 studies demonstrated a strong association between childhood behavioral problems and mental health concerns (e.g., anxiety) in adulthood, with elevated behavioral symptomology being more strongly linked to mental health concerns compared to behavioral diagnoses (Mulraney et al., 2021). Childhood and adolescent behavioral problems have also been linked to physical health problems (Nikstat and Riemann, 2020; Morales-Muñoz et al., 2023), substance use disorders across the lifespan (Mathias et al., 2015), and lower socioeconomic attainment (e.g., lower income) in adulthood (Vergunst et al., 2019). Moreover, all these outcomes are linked; individuals experiencing mental health concerns are at increased risk of physical health conditions or lower socioeconomic attainment, and vice versa (Mezzina et al., 2022). Given the alarming, persistent, and negative impacts of children’s behavioral problems on quality of life (Jonsson et al., 2017), and that the COVID-19 pandemic has led to increased rates of behavioral problems across the globe (Joo and Lee, 2022; Theberath et al., 2022), the research investigating the development of children’s behavioral problems must be addressed as a public health research priority so that timely and effective interventions are designed and implemented.

### Behavioral problems and sex-dependent outcomes

The etiology of children’s externalizing and internalizing problems appears to be sex-dependent (Schore, 2017; Baillargeon et al., 2007; Hill et al., 2017), with some evidence pointing toward a greater risk of both types among male SAAB children (Letourneau et al., 2019; Li et al., 2024; Khan and Avan, 2020; Sarfnaz et al., 2012; Sun et al., 2025). This could be attributable to differences in pregnancy conditions based on child sex (e.g., more testosterone in male child pregnancies) (Finegan et al., 1989; Körner et al., 2019; Rotem et al., 2021), Y-linked chromosomal contributions (Kopsida et al., 2009; Loke et al., 2015), or diagnostic bias that is geared toward better detection among male children (Slobodin and Davidovitch, 2019; Cruz et al., 2024). On the other hand, other researchers have identified a sex-specific etiology whereby SAAB female children are more likely to develop internalizing problems (Gutman and McMaster, 2020) whereas SAAB male children develop externalizing ones (Schore, 2017; Baillargeon et al., 2007; Hill et al., 2017). Genetic researchers using polygenic risk scores have revealed that nearly one-third of the variance of behavioral problems is inherited, with externalizing problems showing greater heritability (Nikstat and Riemann, 2020; Hicks et al., 2004; De Francesco et al., 2024). However, findings suggest that genetic effects associated with children’s behavioral problems may help to explain more of the variance in behavioral stability, whereas social-environmental effects explain more of the variance in behavioral change (van Beijsterveldt et al., 2003; Bartels et al., 2004; Haberstick et al., 2005). Perhaps the social context in which children reside in is therefore important to consider vis-à-vis behavioral change over time (Hannigan et al., 2018). For example, gender stereotypes that girls should not act out whereas boys must be strong and not cry can dictate behavioral expectations and contribute to different behavior problem profiles among female and male SAAB children (Halim et al., 2017). Considering child SAAB when examining the direct and indirect effects of mothers ACEs on their preschool children’s internalizing and externalizing problems is therefore important to identify sex-specific outcomes.

### Covariates relevant to children’s behavioral problems

Other variables are known to impact children’s behavior, including low birthweight, gestational age, racialized status, total household income, mothers’ level of education, mothers’ mental health, and maternal age (Carter et al., 2010). Children born preterm or those born at low birthweight can experience incomplete brain development with smaller brain volumes and atypical neurological structures, leading to developmental delays which increase the risk of behavioral problems (Arpi and Ferrari, 2013). Some findings indicate that racialized children exhibit more behavioral problems than their non-racialized counterparts (Flink et al., 2012), likely attributable to discriminatory access to social determinants of health and healthcare (Cooke et al., 2022), although others report more behavioral problems among white children (Carter et al., 2010). Families reporting lower socioeconomic attainment (i.e., low annual household income, living in poverty, low educational attainment) may struggle to provide optimal care and resources to their children as they experience inequitable opportunities that undermine quality of life (Carter et al., 2010; Kaiser et al., 2017; Tamura et al., 2020). Moreover, mothers’ mental health, including the presence of anxiety and depressive symptoms, are strong predictors of behavioral problems in their children (Franzoi et al., 2024), while mothers’ social support can serve to buffer the effects of mothers’ mental health symptoms (Thomas et al., 2017; Angley et al., 2015). Studies also reveal that children with older mothers have better behavioral outcomes after early infancy than those with younger mothers (Tearne, 2015). Since ACEs are closely linked to all these factors (Nelson et al., 2020; Madigan et al., 2023; Bruner, 2017; Bernard et al., 2022), it is important to include and control for their effects, if possible, when exploring the impacts of ACEs on children’s behavioral problems.

### Purpose of the study

The primary purpose of this study was to explore whether mothers’ adult attachment pattern (continuous variable ranging from secure to insecure) mediates the relationship between their ACEs and children’s internalizing and externalizing behaviors, considering the moderating role of children’s SAAB. It was hypothesized that: (1) ACEs would indirectly increase children’s behavioral problems through more insecure maternal adult attachment pattern and (2) male SAAB children would exhibit more behavioral problems than their female SAAB counterparts. If mediation was observed, post-hoc analysis was planned to examine the nature of the insecure adult attachment pattern (i.e., dismissive and preoccupied adult attachment subtypes).

## Methods

### Study design

In this exploratory correlational study, data from the longitudinal, prospective APrON Study were used (Letourneau et al., 2022). Ethics approval was attained from the University of Calgary Health Research Ethics Board (REB14-1702), University of Alberta Health Research Ethics 11 Biomedical Panel (Pro00002954), and Mount Royal University Human Research Ethics Board (#102823). Informed consent was obtained from all participants prior to data collection at enrollment with updated consent obtained at 5 years.

### Sample and inclusion criteria

Participants were included if they resided within or in proximity to Calgary or Edmonton and were able to attend appointments at the Universities of Calgary or Alberta, were at least 16 years of age or older, were able to speak and read English, were less than 27 weeks gestation at the time of entry into the study, were biological mothers of their children, and remained in the region until at least 3 months postpartum. Women who could not speak English or who planned to relocate from the region were excluded from this study. Data were collected during each trimester of pregnancy and at 3, 6, 12, 24, 36, and 60 months postpartum. The data associated with this study have not been deposited into a publicly available repository; however, the data will be made available on reasonable request to the corresponding author. Additional information regarding APrON can be found elsewhere (Letourneau et al., 2022).

### Participant information

Demographic information for the sample (*n* = 636) is provided (Table 1). Mothers primarily self-identified as white with an undergraduate university degree and a total annual household income greater than $70,000/year. The proportions of female and male (SAAB) children were similar (Table 1).

**Table 1:** Demographic information for participants (*n* = 636).

Demographic information	Proportion (%)
Total annual household income ($ CAD)	
< 40,000	4.25
40,000–69,999	9.75
70,000–99,999	21.70
> 100,000	64.31
Education level	
Completed post-graduate degree	25.47
Completed university degree	49.84
Completed trade/technical school	17.61
Completed high school diploma	7.08
Self-identified racialized status	
White	89.62
Asian	6.92
Latin American	1.73
Other	1.73
Children’s sex assigned at birth	
Male	51.57
Female	48.43

	Mean (SD)
Age of mother (years)	37.64 (4.02)
Age of child (years)	5.08 (0.15)
Children’s birthweight (g)	3390.08 (506.36)
Children’s gestational age (weeks)	39.32 (1.55)

### Included variables and measures

#### Predictor variable

Mothers’ ACEs were measured cross-sectionally when children were approximately 12 months of age using the Adverse Childhood Experiences Questionnaire, a 10-item self-reported “yes” or “no” questionnaire (see Supplementary Figure S1; Felitti et al., 1998). Although dependent on recall and memory (Felitti et al., 1998), and lacking substantial psychometric information supporting its reliability or validity, the ACEs questionnaire is commonly employed to examine early childhood adversity, with converging results of stronger and positive relationships when predicting various diseases as ACEs increase (Hughes et al., 2017).

#### Outcome variable

Children’s internalizing and externalizing problems were assessed via the Behavioral Assessment System for Children, 2nd edition (BASC-2) at about 60 months of age (Reynolds and Kamphaus, 2004). This tool provides a measure of children’s behavior (i.e., internalizing and externalizing problems) and emotional functioning (Reynolds and Kamphaus, 2004). The BASC-2 is a norm-referenced tool that was standardized using a general population sample which included a clinical sample of American children (Reynolds and Kamphaus, 2004). In the present study, parents completed the questionnaire, which asked about directly observed aspects of their child’s behavior and personality (Reynolds and Kamphaus, 2004). Scores were derived by using standardized t-scores with a mean of 50 and a standard deviation of 10. Scores above 70 are considered clinically significant, while scores between 60 to 70 are deemed at-risk, and those below 60 are considered low risk (Reynolds and Kamphaus, 2004). The BASC-2 has been shown to have high construct and convergent validity and high reliability (Reynolds and Kamphaus, 2004).

#### Mediator variable

Mothers’ adult attachment pattern, rated from secure (low score) to insecure (high score), as well as degree of insecure attachment pattern subtypes (dismissive and preoccupied), were coded from the Experiences in Close Relationships–Short Form (ECR-S) (Wei et al., 2007). This was completed when children were about 60 months of age. The ECR-S is a self-report measure that includes 12 questions rated on a Likert Scale ranging from 1 (strongly disagree) to 7 (strongly agree). Since the ECR-S shows similar psychometric properties compared to the original 36-item ECR measure (Brennan et al., 1998; Brenning et al., 2014; Brugnera et al., 2019), we chose to use the shortened version to limit participant burden. Of the 12 questions, six are relevant to preoccupied and six to dismissive adult attachment patterns, respectively. Mothers’ adult attachment pattern was calculated by summing the preoccupied and dismissive adult attachment pattern scores deriving from the 12 questions. Scores for mothers’ adult attachment pattern therefore ranged from 12 to 84 with lower scores indicating a more secure adult attachment pattern while higher scores indicated a more insecure adult attachment pattern. Scores for the preoccupied and dismissive adult attachment pattern subtypes were derived from their respective six questions and ranged from 6 to 42 (Wei et al., 2007).

The ECR-S demonstrates high validity and reliability in assessing adults’ dismissive and preoccupied adult attachment patterns (Wei et al., 2007). While it has not yet been used in research to assess an overall adult attachment pattern (secure versus insecure), prior work suggests that insecure adult attachment reflects an additive combination of both preoccupied and dismissive adult attachment pattern behaviors (Fraley and Waller, 1998). We calculated a McDonald’s Omega coefficient of 0.87 across all 12 items producing the continuous score of adult attachment pattern, suggesting high internal reliability consistency. Additionally, there was a strong general factor driving most items and two group factors contributing additional variance (although one was much stronger than the other). These findings suggest that while the measure is bidimensional, the total score reasonably measures adult attachment pattern continuously from secure to insecure and is appropriate to use in analysis. Overall, this serves as a foundational study employing the ECR-S to measure mothers’ adult attachment pattern as a continuum of secure to insecure.

### Moderator and covariates

Child SAAB, the moderator of this study, was obtained from parent report and validated by birth record. Covariates (i.e., mothers’ self-identified racialized status, total annual household income, highest maternal educational level attained, mothers’ and children’s ages, children’s gestational age and birthweight, mothers’ depressive symptoms, mothers’ anxiety symptoms, mothers’ social support) were included due to theoretical importance, as opposed to statistical. Mothers’ and children’s ages at the 60-month data collection point were calculated by using birthdates and five-year data collection dates. Mothers’ total annual household income, highest educational level attained, and self-identified racialized status were collected at enrolment through maternal report alongside children’s gestational age and birthweight.

Mothers’ depressive symptoms were measured using the Edinburgh Perinatal/Postnatal Depression Scale (EPDS), a valid and reliable 10-item self-report questionnaire that uses a 4-point Likert scale to produce scores ranging from 0 (no depressive symptoms) to 30 (high levels of depressive symptoms; Cox et al., 1987; Bergink et al., 2011; Cosco et al., 2017). Mothers’ anxiety symptoms were measured using the valid and reliable Anxiety Subscale of the Symptom Checklist 90 (Derogatis and Unger, 2010). The SCL-90 Anxiety Subscale is composed of 10 questions using a 5-point Likert scale (0 = not at all to 4 = extremely), with possible scores ranging from 0 (no anxiety symptoms) to 40 (high levels of anxiety symptoms; Derogatis and Unger, 2010). Mothers’ perceived social support was measured using the valid and reliable Social Support Index (SSQ) from the Community Health Survey, a 4-item instrument assessing informational, emotional, affirmational, and instrumental social support using a 5-point Likert scale (0 = none of the time to 4 = all of the time) to produce a total ranging from 0 to 16; higher scores indicate greater levels of perceived social support (Michalos, 2013, p. 194). Mothers’ depressive and anxiety symptoms and perceived social support were measured across various time points (Supplementary Table S1). To capitalize on the repeated measures data, mean values were used.

### Missing data

Although 1,236 participants provided ACEs data at 1 year of age, only 636 provided data on child behavioral problems at 5 years of age (a 48% attrition rate over 4 years). Some variables, such as mothers’ income and depressive symptoms, had missing data that constituted less than 5% of the overall sample; however, missing data for mothers’ anxiety symptoms constituted more than 5% of the sample (*n* = 48). We assessed missing data patterns for mothers’ anxiety via two methods to determine appropriate approaches for handling missingness. Results from a binary logistic regression with maternal anxiety as a binary outcome (0 = data present, 1 = missing) revealed that only maternal age predicted missingness of anxiety symptom data (*p* < 0.05). However, Little’s Test yielded a significant test statistic (*p* < 0.05), suggesting data were not missing completely at random. Taken together, the missing data for mothers’ anxiety symptoms were deemed consistent with a missing at random (MAR) assumption.

Missing data were addressed using multivariate imputation by chained equations (MICE) with twenty imputations, a commonly employed method for addressing data missing at random (Austin et al., 2021). Because the PROCESS macro does not directly pool estimates across multiply imputed datasets, mediation models were estimated using a single completed dataset. To evaluate the robustness of these findings, additional sensitivity analyses were conducted across five additional imputed datasets. These analyses yielded parameter estimates that were consistent in magnitude and direction with the primary model, indicating that the findings were not dependent on a particular imputed dataset; therefore, results from one completed dataset are reported for clarity.

### Data analysis

Demographic data were described using mean values and standard deviations or proportions to characterize the mothers and children. Descriptive data, including mean values, ranges, and standard deviations, were also provided for the predictors and outcomes. R version 4.2.2 was used to conduct the statistical analysis (R Core Team, 2013). All moderation, mediation, and conditional process analyses were completed using the beta release of PROCESS v5 (Hayes et al., in press). An alpha value of 0.05 was used to determine statistical significance in all analyses. Linearity was tested by using residual versus fitted plots. Skewness and kurtosis were assessed to determine whether the data were normally distributed. Correlations between variables were conducted through three different approaches based on variable types: (1) Spearman’s correlation for associations between continuous and/or ordinal variables, (2) analysis of variance for associations between a categorical and continuous variable, and (3) Chi-Square tests along with Cramer’s V for associations between categorical variables (Prematunga, 2012; Kim, 2017).

#### Primary hypothesis

**Figure 1:**
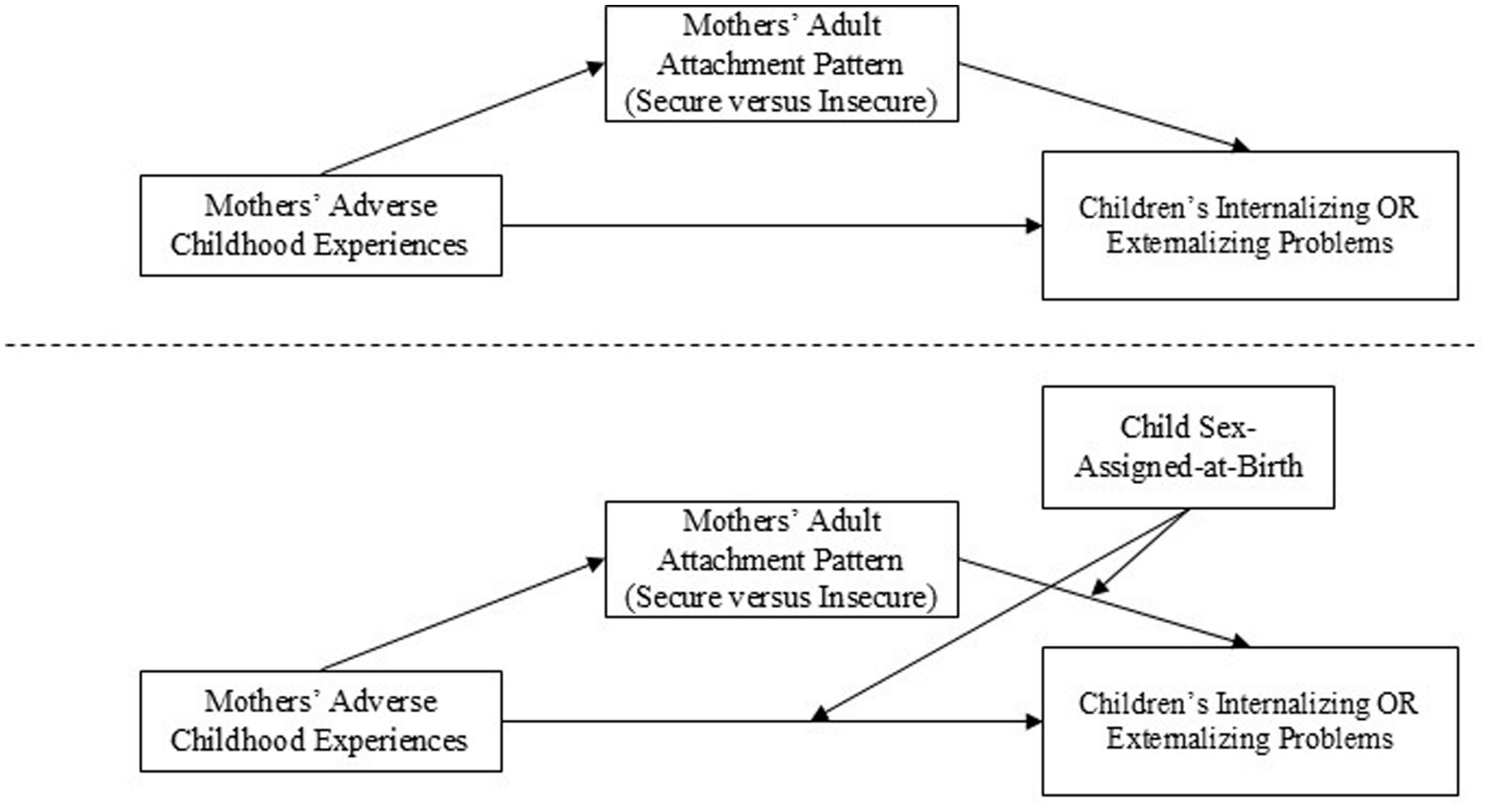
Model template for primary purpose of the study: top panel = simple mediation analysis and bottom panel = conditional process analysis with mediation through mothers’ insecure adult attachment and direct and second-stage moderation by child sex-assigned-at-birth.

To address the primary purpose of the study, a simple mediation model was used to determine the indirect effect of mothers’ ACEs on their preschool children’s internalizing and externalizing problems, respectively, through mothers’ adult attachment pattern (Figure 1, Top Panel). PROCESS v5 implements errors-in-variables regression that debiases the estimation of effects resulting from random measurement error in independent, mediator, and covariate variables in mediation models (Hayes et al., in press). Therefore, based on existing literature, the following conservative Cronbach’s alpha internal reliability coefficients were used in the errors-in-variables routine: 0.70 for ACEs, 0.78 for mothers’ adult attachment pattern, 0.80, 0.85, and 0.90 for mothers’ depressive symptoms, anxiety symptoms, and social support, respectively, and a value of 1.00 for all remaining demographic covariates (i.e., self-identified racialized status, income, maternal educational level, mothers’ and children’s ages, children’s gestational age and birthweight).

A conditional process model was used to determine if the indirect effect of mothers’ ACEs on their preschool children’s internalizing and externalizing problems, respectively, through mothers’ adult attachment pattern was moderated by child SAAB (Figure 1, Bottom Panel). Specifically, child SAAB was included as a direct and second-stage moderator moderation of the pathway from the mediator to the outcome rather than the predictor variable to the mediator; (Hayes, 2017). Child SAAB was also included as a covariate to control for the confounding effects on the first-stage pathways (i.e., pathway from the predictor to the mediator). Again, bootstrapped 95% CI were used to test the indirect effects using 5,000 resamples while direct effects were assessed using *p*-values and 95% CI. Currently, PROCESS does not permit for errors-in-variables regression procedures in models which includes moderators (Hayes et al., in press), therefore random measurement error was not accounted for when conducting conditional process analysis.

#### *Post-hoc* analysis

To address the post-hoc investigation, individual scores of dismissive and preoccupied adult attachment patterns were used. A parallel mediation model was used to determine the indirect effect of mothers’ ACEs on their preschool children’s internalizing and externalizing problems through mothers’ preoccupied and dismissive adult attachment patterns. By inputting mothers’ preoccupied and dismissive adult attachment patterns as parallel mediators, the effect of one mediator was controlled for when estimating the effect of the other. Reliability coefficients from the primary model were again inputted to calculate the errors-in-variables for all independent, mediator, and covariate variables. Bootstrapped CI were used to test the indirect effects using 5,000 resamples while direct effects were assessed using *p*-values and 95% CI.

## Results

### Tests of assumptions

Residual versus fitted plots revealed that the relationship between mothers’ ACEs and internalizing and externalizing problems, respectively, were linear (Supplementary Figures S2, S3). Skewness ranged from −1 to 1 while kurtosis ranged from 1 to 3; although there is discrepancy on what constitutes an acceptable range, particularly for kurtosis (Kim, 2013; Mishra et al., 2019), findings suggested that the data in this study were relatively normally distributed.

### Descriptive statistics of study variables

Low mean values for mothers’ adult attachment pattern suggest that mothers in this sample exhibited relatively secure adult attachment patterns and related subtypes (Table 2). Children’s total externalizing and internalizing problem T scores were nearly identical in this sample (Table 2). When stratified by sex, female children’s total externalizing and internalizing problem scores were 46.98 (±7.50) and 49.26 (±8.46), respectively, while male children’s total externalizing and internalizing problem scores were 49.40 (±8.56) and 48.59 (±8.66), respectively. No notable differences were visually apparent between mean externalizing and internalizing problem scores of female and male children. Lastly, mothers in this study generally reported low depressive and anxiety symptoms and high social support (Table 2).

**Table 2:** Descriptive statistics for main study and covariate variables.

Study variable	Proportion (%)	Mean (standard deviation)
Predictor		
Maternal Adverse Childhood Experiences (ACEs)		
0	53.62	
1	21.38	
2	12.58	
3	5.82	
4	2.67	
≥5	3.93	
Mediator		
Mean adult attachment pattern		28.43 (10.12)
Preoccupied adult attachment pattern		16.19 (6.18)
Dismissive adult attachment pattern		12.24 (5.98)
Outcome		
Internalizing behavioral problems		48.92 (8.56)
Externalizing behavioral problems		48.23 (8.15)
Covariates		
Anxiety symptoms		2.32 (0.26)
Depressive symptoms		4.60 (3.00)
Social support		14.33 (1.98)

### Correlation matrix

Mothers’ ACEs were correlated with their children’s internalizing problems (*r* = 0.09, *p* < 0.03), but only trended towards significance for externalizing problems (*r* = 0.07, *p* < 0.09). Mothers’ ACEs were correlated with mothers’ adult attachment pattern (*r* = 0.25, *p* < 0.01). Also, mothers’ depressive symptoms, anxiety symptoms, and social support were correlated with mothers’ ACEs, mothers’ adult attachment pattern, and children’s internalizing and externalizing problems (all *p* < 0.01). Correlations between all other included variables are also provided (Supplementary Table S2).

#### Primary purpose: mediation through mothers’ adult attachment pattern x child SAAB

An indirect effect of mothers’ ACEs on their children’s externalizing problems through mothers’ adult attachment pattern was supported (effect = 0.31, bootstrap 95% CI [0.11, 0.62]). Similarly, an indirect effect of mothers’ ACEs on their children’s internalizing problems through mothers’ adult attachment pattern was supported (effect = 0.30, bootstrap 95% CI [0.10, 0.58]). Mothers’ ACEs were positively associated with their adult attachment pattern, which in turn, was positively associated with their children’s externalizing and internalizing problems. Mothers’ ACEs did not have a direct effect on their children’s externalizing (effect = −0.15, *p* > 0.67, 95% CI [−0.84, 0.54]) or internalizing (effect = −0.03, *p* > 0.92, 95% CI [−0.71, 0.64]) problems.

When including child SAAB as a direct and second-stage moderator in the model, indirect effects of mothers’ ACEs on their male (effect = 0.31, bootstrap 95% CI [0.15, 0.50]) and female (effect = 0.28, bootstrap 95% CI [0.13, 0.46]) SAAB children’s externalizing problems via mothers’ adult attachment pattern were supported. Mothers’ ACEs positively predicted their adult attachment pattern, which in turn, positively predicted their female and male SAAB children’s externalizing problems. However, the index of moderated-mediation was statistically insignificant (index = −0.03, bootstrap 95% CI [−0.22, 0.16]), indicating that the difference between the two conditional indirect effects was not statistically significant. The interaction between child SAAB and ACEs in predicting children’s externalizing problems was not statistically significant (effect = 0.53, *p* > 0.22); no direct effects of mothers ACEs on male (effect = −0.19, *p* > 0.51, 95% CI [−0.78, 0.39]) or female (effect = 0.33, *p* > 0.29, 95% CI [−0.29, 0.96]) children’s externalizing problems were observed.

Similarly, indirect effects of mothers’ ACEs on their male (effect = 0.35, bootstrap 95% CI [0.19, 0.56]) and female (effect = 0.30, bootstrap 95% CI [0.14, 0.50]) SAAB children’s internalizing problems via mothers’ adult attachment pattern were supported. Mothers’ ACEs positively predicted their adult attachment pattern, which in turn, positively predicted their female and male SAAB children’s internalizing problems. However, the index of moderated-mediation was statistically insignificant (index = −0.05, bootstrap 95% CI [−0.25, 0.13]), indicating that the difference between the two conditional indirect effects was not statistically significant. The interaction between child SAAB and ACEs in predicting children’s internalizing problems was not statistically significant (effect = 0.50, *p* > 0.86, 95% CI [−0.83, 0.98]); no direct effects of mothers ACEs on male (effect = 0.23, *p* > 0.45, 95% CI [−0.39, 0.85]) or female (effect = 0.31, *p* > 0.35, 95% CI [−0.35, 0.97]) SAAB children’s internalizing problems were observed.

#### *Post-hoc* exploration

Since mothers’ adult attachment pattern mediated the association between mothers’ ACEs and their preschool children’s internalizing and externalizing problems, post-hoc analyses were conducted to quantify indirect effects through preoccupied and dismissive adult attachment patterns. Indirect effects of mothers’ ACEs on their children’s externalizing behavioral problems were not supported for either maternal preoccupied (effect = 0.14, bootstrap 95% CI [−0.02, 0.38]) or dismissive (effect = 0.11, bootstrap 95% CI [−0.04, 0.37]) adult attachment pattern. Also, mothers’ ACEs did not have a direct effect on their children’s externalizing problems (effect = −0.15, *p* > 0.67, 95% CI [−0.84, 0.54]).

The indirect effect of mothers’ ACEs on their children’s internalizing behavioral problems was not supported for maternal dismissive adult attachment pattern (effect = 0.07, bootstrap 95% CI [−0.03, 0.26]), while the indirect effect of maternal preoccupied adult attachment pattern was supported (effect = 0.21, bootstrap 95% CI [0.04, 0.47]). Mothers’ ACEs positively predicted their preoccupied adult attachment pattern, which in turn, predicted their children’s internalizing problems. However, a test of differences indicated no statistical difference between the indirect effects (effect = −0.14, bootstrap 95% CI [−0.44, 0.14]). Mothers’ ACEs did not have a direct effect on their children’s internalizing problems (effect = −0.01, *p* > 0.97, 95% CI [−0.69, 0.67]).

## Discussion

The primary aim of the present study explored whether mothers’ adult attachment pattern mediated the association between their ACEs and children’s internalizing and externalizing problems, with consideration of child SAAB. The findings of the present study were consistent with most of the findings of previous research which support the positive association between mothers’ ACEs and their preschool children’s behavioral problems (Cooke et al., 2021). Mothers’ adult attachment pattern mediated the association between mothers’ ACEs and their children’s externalizing and internalizing problems, such that a more insecure adult attachment pattern was associated with more behavioral problems, but no SAAB-specific outcomes were observed. Post-hoc analysis revealed that more insecure preoccupied adult attachment pattern, but not dismissive, among mothers positively mediated the association between mothers’ ACEs and their children’s internalizing, but not externalizing, problems. However, the effect was not statistically different from that of dismissive adult attachment pattern; therefore, we cannot conclude that the effect of preoccupied adult attachment is stronger or different than that of dismissive adult attachment. No indirect effects were observed through dismissive and preoccupied adult attachment pattern for externalizing problems.

Since the sample consisted of more higher socioeconomic homogeneity in terms of maternal self-identified racialized status, educational attainment, and household income, the findings of this study are discussed through a population health framework when warranted. A population health framework considers the various environmental, social, economic, political, and cultural factors and forces which drive health outcomes among specific groups (Chan et al., 2024); by applying such a framework, the protective effects of socioeconomic factors can be speculated upon when considering the associations between mothers’ ACEs, mothers’ adult attachment patterns, and preschool children’s behavioral problems.

This appears to be the first study to employ a total continuous score of adult attachment pattern (secure to insecure) using the ECR-R. While some argue that adult attachment pattern should be categorically measured as per each type (i.e., dismissive and preoccupied), others indicate that individuals’ adult attachment security varies continuously and is multi-dimensional (e.g., has crossovers within and between different categories of attachment) (Fraley et al., 2015). Fraley et al. (2015) argued that the categorization of individuals into specific adult attachment patterns can overlook the nuanced nature of individuals’ differences in relation to attachment behavior. In fact, it was revealed that dismissive and preoccupied adult attachment patterns were indicative of underlying continuous, rather than categorical, dimensions of attachment across different types of relationships via taxometric analyses (Fraley et al., 2015). Moreover, adult attachment pattern is considered an additive combination of characteristics from both preoccupied and dismissive adult attachment patterns (Fraley and Waller, 1998; Griffin and Bartholomew, 1994; Fraley et al., 2015), highlighting the need to examine adult attachment pattern as a continuum of secure to insecure adult attachment pattern. While preoccupied and dismissive adult attachment patterns were examined separately post-hoc, mothers’ overall adult attachment pattern may be a more important explanatory variable that pays respect to differences in adult attachment behaviors while helping to explain the variation in children’s behavioral problems in the context of mothers’ ACEs. For this reason, a total score of adult attachment pattern was derived from the ECR-R by summing the preoccupied and dismissive adult attachment pattern subtypes scores, thus appearing to serve as the first study to employ the ECR-R to measure mothers’ adult attachment pattern across a continuum from secure to insecure.

### Maternal ACEs and mediation through adult attachment patterns

Supporting what was hypothesized, mothers’ adult attachment pattern mediated the association between mothers’ ACEs and their preschool children’s behavioral problems. More specifically, mothers’ ACEs predicted a more insecure adult attachment pattern, which in turn, predicted more externalizing and internalizing problems among their preschool children. Though using a different measure, Roth et al. (2021) reported similar findings, observing an indirect effect of mothers’ ACEs on their children’s behavioral problems via less secure maternal adult attachment. It is speculated that mothers with more insecure adult attachment pattern are more likely to experience greater doubts about their capacities as not only a partner, but also as a parent and individual, leading to unhealthy representations of oneself, poorer emotional regulation, and lower quality interpersonal relationships (Mikulincer and Shaver, 2012). Consequently, children are less likely to receive sensitive, responsive, and nurturing parenting, exacerbating their risk of developing behavioral problems (Lilley et al., 2020; Macfie and Swan, 2009; Zimmer-Gembeck et al., 2022; Gallagher et al., 2015). Roth et al. (2021) appear to be the only others to have specifically examined mothers’ adult attachment pattern as a mediator of the association between mothers’ ACEs and their preschool children’s behavioral problems. Nevertheless, various studies support that mothers’ insecure adult attachment patterns interfere with parenting and child development (Kohlhoff et al., 2024; Cowan et al., 1996; Kim et al., 2019; Ferreira et al., 2024). Taken together, there is compelling evidence that adult attachment pattern plays an important role in the etiology of children’s behavioral outcomes in the context of maternal ACEs.

Post-hoc exploration revealed an indirect effect of mothers’ ACEs on their preschool children’s internalizing problems via a preoccupied adult attachment pattern. An indirect effect was not noted for externalizing problems, and no indirect effects via dismissive adult attachment pattern were supported. However, further exploration revealed that the indirect effect of mothers’ preoccupied adult attachment pattern on children’s behavioral problems was not statistically different from dismissive adult attachment pattern. This suggests that while preoccupied adult attachment pattern may mediate the association between mothers’ ACEs and their children’s internalizing problems, the effect does not differ significantly from that of dismissive adult attachment pattern. In their study, Roth et al. (2021) observed no mediation through either mothers’ preoccupied or dismissive adult attachment patterns. On the other hand, Cooke et al. (2019) observed mediation of the association between mothers’ ACEs and their preschool children’s internalizing and externalizing behavioral problems through preoccupied adult attachment pattern, while a dismissive adult attachment pattern only mediated the association through sequential mediation pathways that included maternal depressive symptoms. This suggests that mothers’ adult attachment pattern measured continuously from secure to insecure, and perhaps preoccupied adult attachment, but not dismissive adult attachment, predict children’s behavioral problems in the context of maternal ACEs in Western contexts. It also supports that mothers’ adult attachment pattern as an additive construct of both preoccupied and dismissive adult attachment patterns may be more indicative of children’s behavioral outcomes, though this requires further exploration.

A preoccupied adult attachment pattern is “thought to reflect individual differences in the way in which people monitor and appraise the availability and accessibility of attachment,” while a dismissive adult attachment pattern is “thought to reflect variation in the way in which people regulate attachment-related thoughts, feelings, and behavior” (Fraley et al., 2015, p. 27). Mothers who exhibit preoccupied adult attachment pattern may exhibit worrying about their intimate partner’s commitment to the relationship, as opposed to regulating their attachment to their partner, unintentionally neglecting their children’s needs, using their children as outlets of support, or displaying an inconsistent mix of both responses (Mikulincer and Shaver, 2012). Consequently, children may experience decreased felt security, which in turn, exacerbates the risk of developing behavioral problems (Ryan et al., 2007). As such, children may instinctively learn to internalize their emotions and feelings, leading to internalizing behavioral problems (Mikulincer and Shaver, 2012). Such effects could be transmitted as early as the prenatal period through fetal programming mechanisms (Glover, 2015). Mothers who experience anxiety-related symptoms due to their perceived or actual lack of partner’s presence can expose the growing fetus to high levels of cortisol, which in turn, increases the child’s length of exposure to the effect of preoccupied attachment and exacerbates the likelihood for cortisol-induced internalizing problems to emerge (Glover, 2015). On the other hand, adults with dismissive adult attachment patterns often report lower anxiety levels than preoccupiedly attached adults, which may allow for mothers who are attached in a dismissive pattern to still provide sufficient care to their child (Cooke et al., 2019). Also, the expected lower levels of anxiety during pregnancy among mothers with dismissive adult attachment patterns compared to those with preoccupied adult attachment patterns suggests less impacts on the fetus during pregnancy (Read et al., 2018). Taken together, this may explain the observed mediation through preoccupied adult attachment in Cooke et al.’s (2019) study, and initially in ours, but also the null results vis-a-vis mediation through dismissive adult attachment pattern observed in both studies.

Our findings also align more broadly with cultural understandings, whereby the association between preoccupied adult attachment pattern and children’s behavioral problems is, at times, more consistent in Western contexts as opposed to some non-Western ones (Roth et al., 2021; Marchand et al., 2004; Walsh and Zadurian, 2023). Through this cultural perspective, it is possible that preoccupied adult attachment is (1) more prevalent in certain Western regions, but not always, due to cross-cultural differences in the perception and expectations of relationships (Agishtein and Brumbaugh, 2013) and (2) more impactful on children than dismissive adult attachment given the predominant impacts preoccupied adult attachment has on various outcomes (Sagone et al., 2023). Nevertheless, more studies are needed to strengthen the evidence on the hypothesized impact of mothers’ preoccupied adult attachment pattern on their preschool children’s behavioral problems between different cultural contexts.

### Child sex did not moderate the maternal ACE-child behavior association

Opposite to what was hypothesized, child SAAB did not directly or indirectly interact with mothers’ ACEs and adult attachment patterns, respectively, to predict more internalizing or externalizing problems among male children. This is inconsistent with previous research, which posits that male children tend to develop more behavioral problems than females (Schore, 2017; Baillargeon et al., 2007; Hill et al., 2017). For example, while not considering the influence of mothers’ ACEs or adult attachment patterns, Baillargeon et al. (2007) found a higher prevalence of externalizing behaviors among preschool boys with 5% exhibiting frequent aggression compared to 1% of girls. These sex-dependent outcomes may be linked to differences in brain maturation rates (Schore, 2017), but also to societal and familial narratives that adhere to binary gender constructs and encourage sex-specific beliefs and behaviors (Brown and Jernigan, 2012; Paul Halpern and Perry-Jenkins, 2016; King et al., 2021). This is supported by other research that shows that children are less likely to endorse traditional gender role attitudes or behaviors when their parents have more egalitarian values and roles within and outside the household (Paul Halpern and Perry-Jenkins, 2016). Since approximately 75% of mothers in this study had attained at least a postsecondary education, these mothers may hold and apply more progressive views on gender roles in their families (Marks et al., 2009), which in turn, may reduce parenting behaviors based on their children’s gender and gender-specific behavioral problems from emerging (Paul Halpern and Perry-Jenkins, 2016; Corley et al., 2022). Higher maternal education has also been associated with lower occurrence of behavioral problems more broadly in their children (Gautam et al., 2023). Future research could consider investigating parental perceptions of gender roles and socioeconomic factors and applying them as additional mediators or moderators of associations between mothers’ ACEs and their children’s behavioral problems.

Not only did most mothers report high educational attainment, nearly two-thirds also reported an annual household income greater than $100,000 CAD. Higher income entails more privileges for families that can optimize children’s behavioral outcomes (Poulain et al., 2019). Children enrolled in higher quality childcare programs, which costs more than typical childcare programs, often exhibit less behavioral problems compared to children enrolled in lower quality programs (Votruba-Drzal et al., 2010). Parents who report higher socioeconomic attainment are also more likely to encourage and enroll their children in extracurricular activities, which has been associated with better outcomes such as academic performance (Tompsett and Knoester, 2023). For example, a meta-analysis conducted by Owen et al. (2022) using data from high-income countries revealed that children and adolescents were more likely to be enrolled in sports when their families resided in higher socioeconomic conditions; this in turn is important given that physical activity is attributed to less behavioral problems in children (Bull et al., 2020). Moreover, participation in extracurricular activities such as sports by female and male children becomes more equal as income increases (Clark, 2014). Therefore, in addition to parental perceptions about gender and their children’s engagement within society, the privileges that children experience residing in higher socioeconomic conditions must also be considered when contextualizing the sex-specific etiology of behavioral problems within different samples.

### Maternal ACEs and their association with children’s behavioral problems

As expected, mothers’ ACEs correlated with their children’s internalizing problems, but contrary to other literature (Arnold et al., 2023), only trended towards statistical significance with children’s externalizing problems. Exactly 75% of the sample experience one ACE or less, suggesting that mothers in this study experienced relatively stable and secure household environments when growing up (Felitti et al., 1998). Also, low T scores on BASC-2 internalizing and externalizing problems composites indicate that overall (Hosokawa and Katsura, 2018), children in this sample were at low risk of behavioral concerns (Reynolds and Kamphaus, 2004; Gautam et al., 2023). This could explain the non-, but trending towards significance, association between mothers’ ACEs and their preschool children externalizing problems. When considering the intergenerational continuity of parenting phenomenon, the lack of ACEs that mothers in this study experienced suggests that mothers were more likely to be exposed to better household environments, which they then fostered themselves as parents (Lomanowska et al., 2017). Moreover, most of the mothers self-reported low levels of anxiety and depressive symptoms (covariates of this study), suggesting high resilience to mental health concerns, and in turn, a greater capacity to engage in more optimal parenting (Honda et al., 2023). Again, when considering the high total annual household income and educational attainment of mothers in this study, it can be presumed that mothers in this sample had more access to goods, resources (e.g., medical interventions), and supports, which helped attenuate mental health symptomology (Goyal et al., 2010; Barakat and Konstantinidis, 2023). Alberta, the province from which this sample is drawn, also offers universal access to healthcare, which promotes access to much needed medical services and treatments (Martin et al., 2018). Therefore, mothers and their preschool children in this sample were exposed to numerous protective factors that could help reduce the impacts of mothers’ ACEs on their children’s behavioral problems.

Findings from studies show that populations who experience more vulnerabilities, such as those experiencing poverty, racialized groups, and females, experience greater levels of ACEs (Madigan et al., 2023; Mersky et al., 2021). For example, Hatch et al. (2020) reported an alarmingly high prevalence of 4 or more ACEs (41%) among a sample of low-income, Black mothers. However, in their study, Hatch et al. (2020) also demonstrated how additional factors, such as moderate-to-high levels of familial social support, can prevent mothers’ ACEs from impacting their children’s behavioral outcomes in samples that experience greater numbers of vulnerabilities. In studies focused on sexual and gender minority individuals, the prevalence of ACEs is usually high, reflecting the increased vulnerability that sexual and gender minority individuals often experience as children (Schnarrs et al., 2023; Dorri et al., 2023). Those experiencing disabilities, mental health concerns, and/or substance use disorders also report a higher prevalence of ACEs (Madigan et al., 2023). Therefore, future research should focus on recruiting more socioeconomically, racially, and sexually and/or gender diverse samples. Such participant samples would perhaps evidence a higher prevalence of behavioral problems among children, which may be linked to the greater prevalence of ACEs and health inequities (Madigan et al., 2023; Schnarrs et al., 2023; Dorri et al., 2023). The high socioeconomic homogeneity of this sample in the present study ultimately limits generalizability, warranting caution when attempting to apply our findings to more heterogenous samples consisting of families experiencing diverse vulnerabilities.

### Implications for practice and research

Mothers’ adult attachment pattern measured continuously from secure to insecure showed strong effects on children’s behavioral problems, whereas an indirect effect was only observed through preoccupied adult attachment on internalizing problems; however, this indirect effect was not statistically significantly different than that through dismissive adult attachment pattern. Although adult attachment pattern is often categorized into different subtypes, findings from this study underscore the importance of measuring adult attachment as a continuous variable from secure to insecure in the context of child health research and practice. Serving as a foundational study, we provide preliminary evidence of using the ECR-R to measure adult attachment pattern continuously from secure to insecure, with evidence of high internal reliability consistency and construct validity, urging other researchers to consider doing the same. Researchers should also examine moderators and mediators of associations between adult attachment pattern (measured from secure to insecure) and children’s behavioral problems, whether directly or indirectly, to identify tangible factors on which to act upon to promote more secure attachment. Healthcare providers, such as psychologists, can first use the continuous measure of adult attachment pattern to gauge a parent’s attachment security. Subsequently, they can identify attachment-related behaviors on which to focus on, regardless of whether they reflect preoccupied or dismissive adult attachment pattern behaviors. However, this means that healthcare providers must be equipped with knowledge of the multidimensional features of adult attachment pattern so that they can best address various insecurely attached behaviors.

Since correlations between mothers’ ACEs and their children’s behavioral problems were partially supported, findings reveal potential associations, especially for internalizing problems. Moreover, no conditional effects were observed by child SAAB. Alluding to the fact that other factors likely help to describe the maternal ACE-child behavioral problem association, findings support adult attachment pattern as a tangible factor upon which to act on to reduce the intergenerational effects of maternal ACEs and children’s behavioral problems, especially those which are internally directed, with no specificity related to child SAAB.

### Limitations and strengths

It is important to note that the participants in this study consisted largely of self-identified, highly educated, white mothers with high incomes and low exposure to ACEs which limits generalizability. Also, selective attrition bias likely occurred among those reporting lower education and income due to the nature of prospective longitudinal cohorts (Larsson, 2021). However, this can also be considered a strength as it increases generalizability to groups living in similar sociopolitical contexts with high socioeconomic attainment. Some may view the use of a total score from the ECR-R to measure adult attachment pattern continuously from secure to insecure as a limitation in this study; we view it as a strength, serving as a foundational study which can pave the way the ECR-R is used in future research and clinical settings. The clear exposure-outcome relationship means that an association can be discussed.

## Conclusion

The findings of this study reinforce that mothers’ ACEs are associated with their preschool children’s behavioral problems, emphasizing the long-term intergenerational impact of early adversity. Moreover, this study is the first to employ the ECR-R to measure mothers’ adult attachment pattern across a continuum from secure to insecure. While prior research has established a link between maternal ACEs and children’s behavioral problems, this study reinforces the potential for adult attachment pattern insecurity to serve as a key mechanism in this process. Findings allude to the importance of conceptualizing mothers’ adult attachment pattern as a continuum of secure to insecure as opposed to only individual subtypes (i.e., dismissive and preoccupied) and the importance of intervening accordingly. The absence of moderation by child SAAB suggests that maternal influences may be particularly strong; it is posited that the high socioeconomic homogeneity of this sample resulted in more egalitarian family dynamics, buffering the emergence of sex-specific behavioral problems. Future research should measure for egalitarian values in families to explore how these dynamics might buffer against the transmission of adversity in more diverse populations. Interventions that target mothers’ adult attachment insecurity could help to prevent and attenuate the impact of mothers’ ACEs on their children’s behavioral outcomes and optimize familial health.

## Data Availability

The raw data supporting the conclusions of this article will be made available by the authors, without undue reservation.
