# Correction: Maternal adverse childhood experiences and preschool children's behavioral problems: exploring mediation via an adapted measure of adult attachment pattern

**DOI:** 10.3389/fpsyg.2026.1832110

**Published:** 2026-04-07

**Authors:** Stefan Kurbatfinski, Aliyah Dosani, Andrew F. Hayes, Deborah Dewey, Nicole Letourneau

**Affiliations:** 1 Department of Community Health Sciences, Cumming School of Medicine, University of Calgary, Calgary, AB, Canada; 2 Alberta Children's Hospital Research Institute (ACHRI), Owerko Centre, Calgary, AB, Canada; 3 Faculty of Health, Community, and Education, School of Nursing and Midwifery, Mount Royal University, Calgary, AB, Canada; 4 O'Brien Institute for Public Health, Cumming School of Medicine, University of Calgary, Calgary, AB, Canada; 5 Haskayne School of Business, University of Calgary, Calgary, AB, Canada; 6 Hotchkiss Brain Institute, Cumming School of Medicine, University of Calgary, Calgary, AB, Canada; 7 Departments of Community Health Sciences and Pediatrics, Cumming School of Medicine, University of Calgary, Calgary, AB, Canada; 8 Faculty of Nursing and Cumming School of Medicine (Departments of Community Health Sciences, Pediatrics, and Psychiatry), University of Calgary, Calgary, AB, Canada

**Keywords:** adult attachment pattern, mediation and moderation, maternal adverse childhood experiences, children's behavior, mental health, child sex-assigned-at-birth, Experiences in Close Relationships

In the published article, the attachment measure was incorrectly referred to as the Revised Experiences in Close Relationships Questionnaire (ECR-R). However, the measure was correctly described as the Experiences in Close Relationships–Short Form (ECR-S) in the Methods section, and the analyses were conducted using the ECR-S. While we originally allude to using the ECR-S, it is unclear. Therefore, the correction involves replacing references to the ECR-R with the Experiences in Close Relationships–Short Form (ECR-S) in the abstract, measures section, and throughout the paper. Also, when citing the ECR-S, the citation needs to be changed from Fraley et al., 2000 to Wei et al., 2007 (the authors who developed the ECR-S), meaning the Fraley et al., 2000 citation should be removed altogether. Similarly, the Sibley et al., 2005 citation is no longer correct in describing validity and reliability of the measure, which should be replaced by Wei et al., 2007. This correction does not affect the results or conclusions of the study.

A correction has been made to the section [Methods, Included Variables and Measures, Mediator Variable, Pages 04-05]:

“Mothers' adult attachment pattern, rated from secure (low score) to insecure (high score), as well as degree of insecure attachment pattern subtypes (dismissive and preoccupied), were coded from the Experiences in Close Relationships–Short Form (ECR-S) (Wei et al., 2007). This was completed when children were about 60 months of age. The ECR-S is a self-report measure that includes 12 questions rated on a Likert Scale ranging from 1 (strongly disagree) to 7 (strongly agree). Since the ECR-S shows similar psychometric properties compared to the original 36-item ECR measure (Brennan et al., 1998; Brenning et al., 2014; Brugnera et al., 2019), we chose to use the shortened version to limit participant burden. Of the 12 questions, six are relevant to preoccupied and six to dismissive adult attachment patterns, respectively. Mothers' adult attachment pattern was calculated by summing the preoccupied and dismissive adult attachment pattern scores deriving from the 12 questions. Scores for mothers' adult attachment pattern therefore ranged from 12 to 84 with lower scores indicating a more secure adult attachment pattern while higher scores indicated a more insecure adult attachment pattern. Scores for the preoccupied and dismissive adult attachment pattern subtypes were derived from their respective six questions and ranged from 6 to 42 (Wei et al., 2007).

The ECR-S demonstrates high validity and reliability in assessing adults' dismissive and preoccupied adult attachment patterns (Wei et al., 2007). While it has not yet been used in research to assess an overall adult attachment pattern (secure versus insecure), prior work suggests that insecure adult attachment reflects an additive combination of both preoccupied and dismissive adult attachment pattern behaviors (Fraley and Waller, 1998). We calculated a McDonald's Omega coefficient of 0.87 across all 12 items producing the continuous score of adult attachment pattern, suggesting high internal reliability consistency. Additionally, there was a strong general factor driving most items and two group factors contributing additional variance (although one was much stronger than the other). These findings suggest that while the measure is bidimensional, the total score reasonably measures adult attachment pattern continuously from secure to insecure and is appropriate to use in analysis. Overall, this serves as a foundational study employing the ECR-S to measure mothers' adult attachment pattern as a continuum of secure to insecure.”

Another error in the published article, the handling of missing data was described as using multiple imputation via the mice package. To clarify, multiple imputation was conducted using mice, and a single imputed dataset was subsequently used in the PROCESS analyses. To ensure that this analytic choice did not influence the findings, additional sensitivity analyses were conducted using alternative imputation specifications. These analyses yielded results consistent with those reported in the original article, with no impacts made on the results or discussion of the paper.

A correction has been made to the section [Methods, Missing Data, Page 05]:

Corrected paragraph: “Although 1,236 participants provided ACEs data at 1 year of age, only 636 provided data on child behavioral problems at 5 years of age (a 48% attrition rate over 4 years). Some variables, such as mothers' income and depressive symptoms, had missing data that constituted less than 5% of the overall sample; however, missing data for mothers' anxiety symptoms constituted more than 5% of the sample (*n* = 48). We assessed missing data patterns for mothers' anxiety via two methods to determine appropriate approaches for handling missingness. Results from a binary logistic regression with maternal anxiety as a binary outcome (0 = data present, 1 = missing) revealed that only maternal age predicted missingness of anxiety symptom data (*p* < 0.05). However, Little's Test yielded a significant test statistic (*p* < 0.05), suggesting data were not missing completely at random. Taken together, the missing data for mothers' anxiety symptoms were deemed consistent with a missing at random (MAR) assumption.

Missing data were addressed using multivariate imputation by chained equations (MICE) with twenty imputations, a commonly employed method for addressing data missing at random (Austin et al., 2021). Because the PROCESS macro does not directly pool estimates across multiply imputed datasets, mediation models were estimated using a single completed dataset. To evaluate the robustness of these findings, additional sensitivity analyses were conducted across five additional imputed datasets. These analyses yielded parameter estimates that were consistent in magnitude and direction with the primary model, indicating that the findings were not dependent on a particular imputed dataset; therefore, results from one completed dataset are reported for clarity.”

The original version of this article has been updated.

