# Supplementary material for: Maternal adverse childhood experiences and preschool children’s behavioral problems: exploring mediation via an adapted measure of adult attachment pattern
Source: Front Psychol. 2025 Sep 2;16:1596613. doi: 10.3389/fpsyg.2025.1596613 (PMC12439478; doi:10.3389/fpsyg.2025.1596613)
Supplement: Supplementary file 1 [file Data_Sheet_1.docx]

| **Variable** | **Time Point** | | | | | | | | |
| --- | --- | --- | --- | --- | --- | --- | --- | --- | --- |
|  | **<13 weeks*** | **14-32 weeks*** | **32 weeks*** | **3 Months** | **6 Months** | **1 Year** | **2 Years** | **3 Years** | **5 Years** |
| Maternal Adverse Childhood Experiences |  |  |  |  |  | 1.02 (+0.08) |  |  |  |
| Children’s Behavioural Problems |  |  |  |  |  |  |  |  | 5.08 (+0.15) |
| Maternal Depressive Symptoms | X | X | X | 0.24 (+0.06) | 0.51 (+0.09) | 1.02 (+0.08) |  |  |  |
| Maternal Anxiety Symptoms | X | X | X | 0.24 (+0.06) | 0.51 (+0.09) | 1.02 (+0.08) |  |  | 5.08 (+0.15) |
| Maternal Social Support | X | X |  | 0.24 (+0.06) |  | 1.02 (+0.08) | 2.04 (+0.21) | 3.07 (+0.63) | 5.08 (+0.15) |
| Maternal Adult Attachment Pattern |  |  |  |  |  |  |  |  | 5.08 (+0.15) |

**Supplementary Table S1.** *Approximate time at which each variable was collected measured in child age in years (mean and SD)*

NOTE: Asterisk * indicates prenatal period

**Supplementary Table S2.** *Correlation matrix between all model variables*

|  | **(1) Maternal ACEs** | **(2) Internalizing Problems** | **(3) Externalizing Problems** | **(4) Adult Attachment Pattern** | **(5) Child SAAB** | **(6) Self-Identified Ethnicity** | **(7) Annual Household Income** | **(8) Maternal Education** | **(9) Maternal Age** | **(10) Child Age** | **(11) Child Birthweight** | **(12) Child Gestational Age** | **(13) Maternal Depressive Symptoms** | **(14) Maternal Anxiety Symptoms** | **(15) Maternal Social Support** |
| --- | --- | --- | --- | --- | --- | --- | --- | --- | --- | --- | --- | --- | --- | --- | --- |
| **1** | - |  |  |  |  |  |  |  |  |  |  |  |  |  |  |
| **2** | 0.09* | - |  |  |  |  |  |  |  |  |  |  |  |  |  |
| **3** | 0.07 | 0.49** | - |  |  |  |  |  |  |  |  |  |  |  |  |
| **4** | 0.25** | 0.26** | 0.23** | - |  |  |  |  |  |  |  |  |  |  |  |
| **5** | 0.07 | n.s. | ** | n.s. | - |  |  |  |  |  |  |  |  |  |  |
| **6** | 0.14 | n.s. | n.s. | n.s. | 0.08 | - |  |  |  |  |  |  |  |  |  |
| **7** | -0.08 | -0.08* | -0.10* | 0.02 | 0.06 | 0.17** | - |  |  |  |  |  |  |  |  |
| **8** | 0.19** | n.s. | n.s. | n.s. | 0.05 | 0.12 | 0.19** | - |  |  |  |  |  |  |  |
| **9** | 0.07 | -0.03 | -0.03 | 0.13** | n.s. | n.s. | 0.17** | ** | - |  |  |  |  |  |  |
| **10** | 0.02 | 0.07 | 0.10* | 0.01 | * | n.s. | 0.02 | n.s. | 0.03 | - |  |  |  |  |  |
| **11** | -0.06 | -0.04 | -0.03 | -0.06 | ** | ** | 0.06 | n.s. | -0.08 | -0.02 | - |  |  |  |  |
| **12** | 0.01 | -0.02 | -0.03 | -0.03 | n.s. | * | -0.04 | n.s. | -0.08 | -0.02 | 0.50** | - |  |  |  |
| **13** | 0.22** | 0.28** | 0.18** | 0.33** | n.s. | n.s. | -0.10** | * | 0.02 | 0.04 | -0.03 | -0.06 | - |  |  |
| **14** | 0.27** | 0.28** | 0.17** | 0.28** | n.s. | n.s. | -0.11** | * | -0.05 | 0.05 | 0.00 | 0.00 | 0.69** | - |  |
| **15** | -0.15** | -0.18** | -0.21** | -0.45** | n.s. | * | 0.04 | n.s. | -0.20** | -0.04 | -0.01 | 0.04 | -0.36** | -0.25** | - |

NOTE: * = p<0.05; ** = p<0.01; blank spaces with asterisks indicate significant ANOVA results; n.s. indicates non-significant ANOVA results.

**Supplementary Figure S1.** *Adverse Childhood Experiences Questionnaire.*

Did a parent or other adult in the household often… Swear at you, insult you, put you down, or humiliate you? or Act in a way that made you afraid that you might be physically hurt?

Did a parent or other adult in the household often… Push, grab, slap, or throw something at you? or Ever hit you so hard that you had marks or were injured?

Did an adult or person at least 5 years older than you ever… Touch or fondle you or have you touch their body in a sexual way? or Try to or actually have oral, anal, or vaginal sex with you?

Did you often feel that… No one in your family loved you or thought you were important or special? or Your family didn’t look out for each other, feel close to each other, or support each other?

Did you often feel that… You didn’t have enough to eat, had to wear dirty clothes, and had no one to protect you? or Your parents were too drunk or high to take care of you or take you to the doctor if you needed it?

Were your parents ever separated or divorced?

Was your mother or stepmother: Often pushed, grabbed, slapped, or had something thrown at her? or Sometimes or often kicked, bitten, hit with a fist, or hit with something hard? or Ever repeatedly hit over at least a few minutes or threatened with a gun or knife?

Did you live with anyone who was a problem drinker or alcoholic or who used street drugs?

Was a household member depressed or mentally ill or did a household member attempt suicide?

Did a household member go to prison?


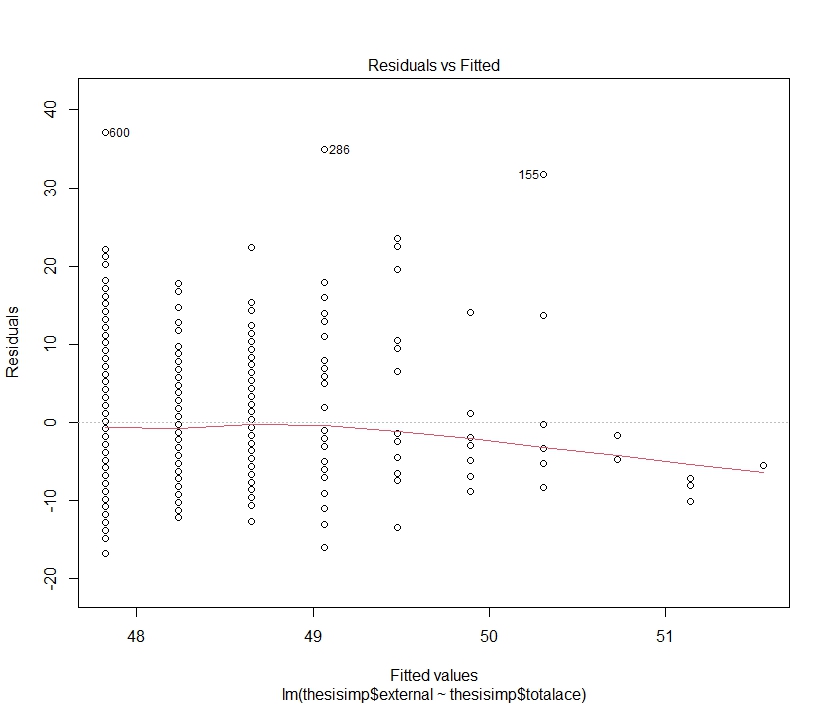


**Supplementary Figure S2.** *Residual versus fitted plot of children’s externalizing problems predicted by maternal ACEs (n=636)*

*
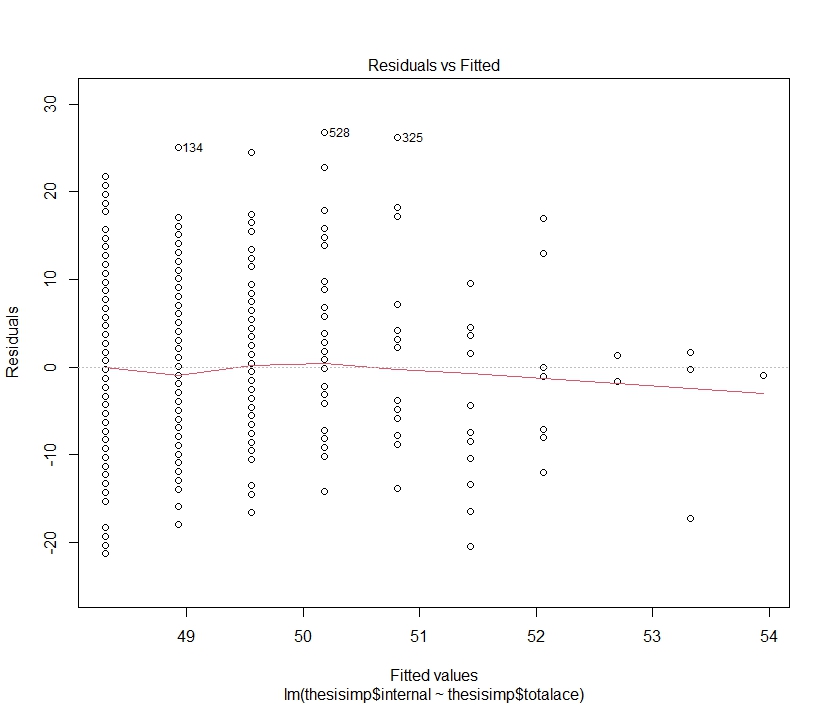
*

**Supplementary Figure S3.** *Residual versus fitted plot of children’s internalizing problems predicted by maternal ACEs (n=636)*
